# Mediterranean White Lupin Landraces as a Valuable Genetic Reserve for Breeding

**DOI:** 10.3390/plants10112403

**Published:** 2021-11-07

**Authors:** Ioannis Zafeiriou, Alexios N. Polidoros, Eirini Baira, Konstantinos M. Kasiotis, Kyriaki Machera, Photini V. Mylona

**Affiliations:** 1 Institute of Plant Breeding & Genetic Resources, HAO-DEMETER, 57001 Thermi, Greece; zafeirioannis@gmail.com; 2 Laboratory of Genetics and Plant Breeding, School of Agriculture, Aristotle University of Thessaloniki, 54124 Thessaloniki, Greece; palexios@agro.auth.gr; 3 Laboratory of Pesticides’ Toxicology, Department of Pesticides Control and Phytopharmacy, Benaki Phytopathological Institute, 8 St. Delta Street, Kifissia, 14561 Athens, Greece; e.baira@bpi.gr (E.B.); k.kasiotis@bpi.gr (K.M.K.); k.machera@bpi.gr (K.M.)

**Keywords:** lupin, Mediterranean, genetic diversity, marker assisted selection, traits, alkaloids, metabolomics

## Abstract

Legumes crops are important for sustainable agriculture and global food security. Among them white lupin (*Lupinus albus* L.), is characterized by exceptional protein content of high nutritional value, competitive to that of soybean (*Glycine max*) and is well adapted to rainfed agriculture. However, its high seed-quinolizidine alkaloid (QA) content impedes its direct integration to human diet and animal feed. Additionally, its cultivation is not yet intensive, remains confined to local communities and marginal lands in Mediterranean agriculture, while adaptation to local microclimates restrains its cultivation from expanding globally. Hence, modern white lupin breeding aims to exploit genetic resources for the development of “sweet” elite cultivars, resilient to biotic adversities and well adapted for cultivation on a global level. Towards this aim, we evaluated white lupin local landrace germplasm from Greece, since the country is considered a center of white lupin diversity, along with cultivars and breeding lines for comparison. Seed morphological diversity and molecular genetic relationships were investigated. Most of the landraces were distinct from cultivars, indicating the uniqueness of their genetic make-up. The presence of *pauper* “sweet” marker allele linked to low seed QA content in some varieties was detected in one landrace, two breeding lines, and the cultivars. However, QA content in the examined genotypes did not relate with the marker profile, indicating that the marker’s predictive power is limited in this material. Marker alleles for vernalization unresponsiveness were detected in eight landraces and alleles for anthracnose resistance were found in two landraces, pointing to the presence of promising germplasm for utilization in white lupin breeding. The rich lupin local germplasm genetic diversity and the distinct genotypic composition compared to elite cultivars, highlights its potential use as a source of important agronomic traits to support current breeding efforts and assist its integration to modern sustainable agriculture.

## 1. Introduction

White lupin (*Lupinus albus* L.) is considered one of the most important domesticated lupin species, when regarding the nutritional value of seeds [1]. Characterized by its protein-rich composition (up to 44% of the total dry mass), a high-quality fatty-acids’ profile, and a plethora of health-promoting bioactive molecules, white lupin denotes a nutritional treasure worthy to be harnessed [2,3]. However, antinutritional compounds like quinolizidine alkaloids (QA) present in high contents in wild white lupin populations and bitter cultivars, reduce its nutritional value and prevent the use of unprocessed seeds for human and animal consumption. From an agronomic perspective, white lupin promotes both N- and P- soil enrichment, through the formation of nodules and proteoid roots [4], and can be cultivated under rainfed intercropping systems, encouraging low-input farming systems [5,6,7]. Therefore, it suggests a promising choice for promoting global food security and environmental protection, through sustainable agriculture [8].

White lupin originates in the southern Balkans, and “graecus-type” natural populations disperse throughout the Eastern Mediterranean basin, where landraces are used for human consumption and fodder, dating back to 2000BC [9]. However, because of its adaptability to marginal regions, and the requirement of a post-harvest debittering process [10], white lupin’s cultivation was restricted mainly to local communities on barren lands. Currently, lupins account for only 1% of the main grain legumes cultivated worldwide. Lupin production and cultivated area worldwide for 2019 was estimated at about 1,006,842 tonnes and 887,111 ha, respectively. Australia is the largest producer, with 47.1% of the global production, while Europe is second (39%) according to FAOSTAT [11]. In Europe *L. angustifolius* L. and *L. luteus* L. are the predominant cultivated species in the north and countries with more than 10,000 ha of lupins are Poland, the Russian Federation, Germany, Belarus, and Ukraine. In the south, where *L. albus* L. is predominant, Italy (5000 ha), France (3600 ha), and Spain (3045 ha) are the main lupin-producing countries [12].

Modern breeding efforts to improve white lupin agronomic characteristics are very recent [9]. Thus, there is still significant variability even among commercial germplasm for the most essential breeding targets, namely, the seed’s nutritional value [13] and the toxic, bitter secondary metabolites QAs [14,15]. The total alkaloid content in white lupin varies from 0.02 to 12.73% of the seed’s dry weight. Cultivars possessing the *pauper* gene contain 0.02–0.05% alkaloids of the seed dry weight [16]. The recently published white lupin pangenome study demonstrated that *pauper* locus has a key role in the species domestication and breeding [17].

Other important agronomic characters of white lupin, which attracted the breeders’ attention, are vernalization insensitivity [18], anthracnose resistance [19], yield stability [20], and abiotic-stress acclimation [21].

White lupin’s global commercial potential has incited the breeding interest to focus not only in yield boost, but also in expanding its cultivation to agroclimatic regions, other than the Mediterranean basin, as extreme climate-change-related phenomena push the cultivation of some crops northwards [22]. On top of that, restricted precipitation levels during spring and frequent dry spells, throughout the Mediterranean basin, as a consequence of climate change [23,24,25], have a detrimental impact on pollen fertility [26], pollinator-flower interactions [27,28], pod filling, and seed development, resulting in premature harvesting and yield losses [29,30]. Vernalization insensitivity and flowering time in white lupin are controlled by a highly complex multi-locus system [18,31].

Anthracnose is a global fungal disease, responsible for devastating epidemics, characterized by significant yield losses [32,33,34]. Temperatures over 10 °C and humid weather promote conidia germination, with 25 °C being optimal for fungal growth; whereas dry summer conditions are favorable for the preservation of inoculum on unharvested plant tissues [35,36]. *Colletotrichum lupini,* is mostly identified as the responsible pathogen for lupin anthracnose. Nonetheless, it has been reported in several other crops such as olive [37]. The pathogen emerges as an alarming polyphagous phytopathogenic strain for the Mediterranean agriculture. White lupin breeding has been directed to the creation of elite anthracnose resistant cultivars, employing map construction, genomic screening, phenotyping tools, field experimentation, and generation of molecular markers, to detect anthracnose resistant accessions [19,38,39,40,41]. Thus far, Ethiopian landraces have been extensively studied, revealing a highly diverse germplasm and embodying unique loci that confer resistance to anthracnose [38,42].

Successful breeding significantly depends on the extent of the available genetic resourcesPhenotypic and molecular markers have already been used in few studies, to estimate the genetic diversity between wild and breeding white lupin germplasm, and to enable incorporation of potentially valuable alleles from distantly related wild accessions to the genetic pool of elite cultivars [43,44]. Additionally, molecular markers are continuously developed, for the effective selection of germplasm with desired traits [31,45]. Furthermore, genomic resources are now available in white lupin and their use will greatly advance our understanding of the species diversity. In such an effort, very recently genome sequences of 39 accessions were used to establish a white lupin pangenome that can be used as resource to identify genes linked to important agronomic traits and analyze genetic variability [17].

Although such progress will inevitably lead to development of more sophisticated tools to explore genetic variation in white lupin genetic resources, up to now SSR markers have been proved integral tools to investigate species diversity. The Balkan Peninsula represents a yet untapped germplasm diversity center for white lupin, concealing potentially valuable loci in landraces and natural populations that could promote adaptability to climate-change-relevant extreme conditions [46,47]. In this study we applied available SSR molecular markers linked to agronomically important traits and morphological seed characteristics to explore the genetic diversity of white lupin Greek landraces and compare it with that observed in commercial varieties and breeding lines. Results of the study may facilitate marker assisted breeding in white lupin and enable identification and introgression of valuable alleles into new elite cultivars.

## 2. Results

### 2.1. Seed Morphological Characters

All seed morphological characters’ measurements were statistically analyzed and a summary of the statistics is shown in Table 1. The Multivariate Analysis of Variance indicated that all 45 accessions are distinguished from one another, when regarding their seed morphology, with statistical significance (*p* = 0.003). A Pearson’s correlation matrix revealed strong correlation between seed area with TSW, perimeter and width (above 0.662) and between mean Gray Value with maximum and median Gray Value (above 0.709), suggesting, that a single character measurement is sufficient to represent highly correlated characters. Circularity showed only small to medium significant correlation with seed width, minimum, and mean and median Gray Value (above 0.323). Integrated density had a negative correlation with the rest of the variables, statistically significant only to height and width.

**Figure 1:**
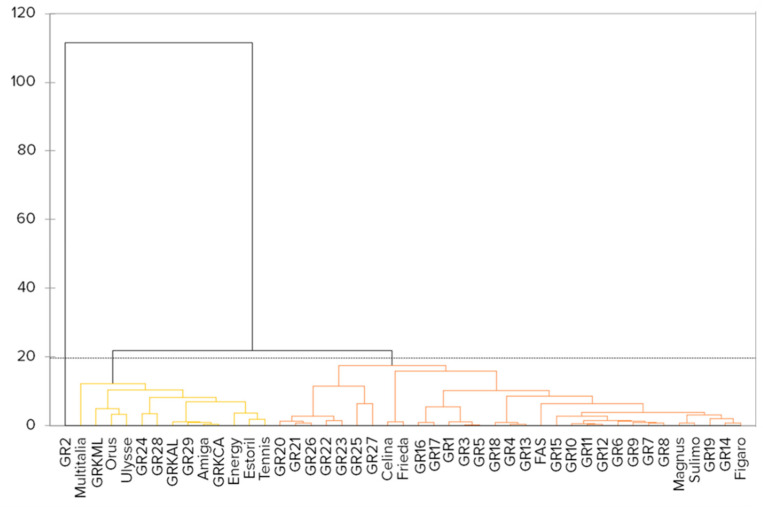
UPGMA dendrogram based on the Euclidean distance of the morphological markers of the white lupin accessions’ seeds.

Partitioning of the variance and grouping of the accessions, regarding seed morphology, were estimated by hierarchical clustering under the Unweighted Pair Group Method with Arithmetic mean (UPGMA) and Principal Component Analyses (PCA). The UPGMA analysis gave formation to a three cladded dendrogram (Figure 1), with most of the landraces grouped together in one clade, along with some of the cultivars that were separated in subclades. A particular landrace, GR2, presenting unique seed morphology formed a single clade alone. A third clade consisted exclusively of cultivars and breeding lines. A PCA performed to calculate the Eigenvalues and variability partitioning to the different vectors, with the first two dimensions explaining 61.92% of the data variability. In a bi-plot graph of the PCA, the genotypes that were found to be highly correlated, are in close proximity with one another, and the distribution of the accessions forms two groups, with GR2 being an outlier (data not shown). Regarding the seed surface, landraces GR24, GR25, GR27, and GR28 have biconcave seeds of orange-white color, with dark flecks and a characteristic black line peripheral to the hilum, while the rest of the accessions have white-yellowish seeds with no spot pattern (online Supplementary Figures S3–S6).

### 2.2. Markers for Genetic Analysis

The molecular marker HRM analysis elucidated 53 distinct allelic patterns, which were assumed as different genotypes. The marker PIC values (Polymorphic Information Content) ranged from 0.1 for GI-F1 to 0.8 for LSSR06a and LSSR41, with an average of 0.5 (online Supplementary Table S1). In order to statistically analyze the results, a binary matrix was constructed, based on the allelic patterns generated from the HRM analysis, where “1” indicate the presence of a specific allelic pattern, and “0” indicate its absence. To that binary matrix the results of the presence or absence of the alleles for anthracnose, vernalization and alkaloid biosynthesis were also incorporated.

**Figure 2:**
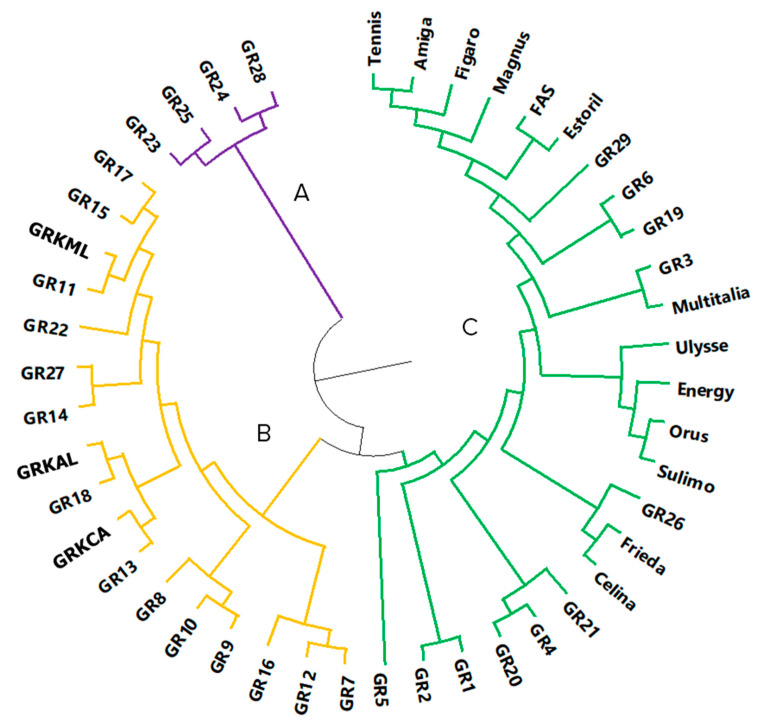
UPGMA dendrogram, clustering the accessions in three main clusters (A, B, and C).

Based upon this matrix, we calculated the Nei’s genetic distance among all samples and formed a circular dendrogram, according to UPGMA clustering (Figure 2). As presented in the dendrogram, the whole germplasm is divided in three major clades. Clade A consists of landraces originated from Andros island, while clade B incorporates most landraces from Northern Greece, Peloponnese, Lemnos island, and Leros island and the three breeding lines. Clade C encloses all the commercial cultivars along with all the landraces from Crete, one landrace from Andros, Lemnos, and Leros islands and one breeding line. Interestingly the Cretan landraces are sub-grouped into two subclades, one housing landraces (GR1, GR2, GR3 and GR20) and the other holding GR3 and Multitalia. Next to the later are landraces GR19 from Crete and GR6 from Leros island. While the breeding line GR29 is grouped together with six commercial cultivars in a subcluster.

**Figure 3:**
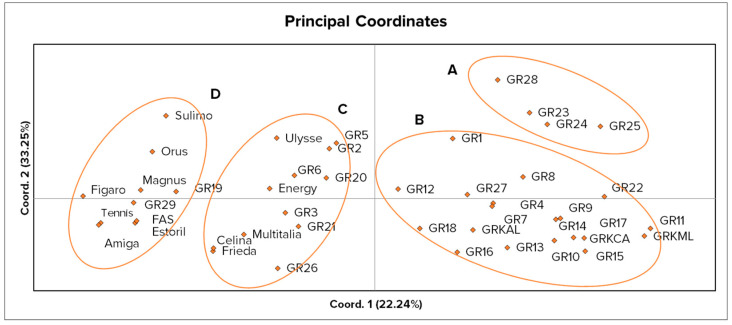
Two-dimensional PCoA analysis of 45 white lupin accessions. Representation of four non-overlapping groups (A–D) delimited by red ovals.

Patterns of genetic relationships among the studied germplasm are visualized by PCoA analysis, with the first two axes explaining the 55.49% of the observed variance (Figure 3). Four distinguishable groups were formed, and genetically similar genotypes are enclosed within the red oval shapes. Genotypes within groups C and D show a narrow distribution on coordinate 1, while genotypes within groups A and B are more broadly distributed, indicating higher genetic variability encompassed within the landraces. The Mantel’s test performed for inspecting the potential correlation among the genetic distances calculated by the seed morphology measurements and by molecular markers showed a non-significant low positive trend (r = 0.082, *p* = 0.068). Possible associations of each morphological trait with the molecular markers were tested by linear regression analysis, but the results did not reveal any significant correlation.

### 2.3. Marker Genotypes Linked to Significant Traits

The 45 accessions under examination were further characterized for presence of marker alleles identified in other studies to be linked to agronomic traits important for white lupin breeding. For resistance to anthracnose, the accessions were screened with TP222136 and TP338761 molecular markers [18]. Most of the landraces, together with cultivars Frieda and Celina, possess the resistance-conferring antr04_1/antr05_1 locus allele, while landraces GR1, GR2, GR23, GR25, and cultivar Sulimo, carry the resistance-conferring antr04_2/antr05_2 locus allele (Table 2). Notably, landraces GR23 and GR25 from Andros island are the only landraces to hold both resistance-conferring alleles.

Regarding vernalization responsiveness, three molecular markers were used, capable of distinguishing between vernalization responsive and non-responsive genotypes, namely SEP3-F1, GI-F1, and FTa1-F1 [18]. Regarding *SEPALLATA 3* locus, all cultivars apart from Sulimo, breeding line GR29, as well as landraces GR3, GR7, GR12, GR16, GR19, GR21, and GR26, bring upon the early flowering allele (Table 2). The *FLOWERING LOCUS T* early flowering allele was present in all accessions, except for five, as in those cases, the aforementioned locus could not be detected using the specific primer set (Table 2). Regarding *GIGANTEA* locus, detection of the different alleles in agarose gel electrophoresis was inconclusive for GI-F1 marker, so the samples were subjected to Sanger sequencing (CeMIA SA, Larissa, Greece) in order to detect the restriction site of *AciI*, which was present in landraces GR5, GR10, and cultivar Frieda (Table 2). In order to investigate the presence of the “sweet” allele on *pauper* locus, LAGI01_35805_F1_R1 molecular marker was employed [48], revealing the probability of a low alkaloid genotype in all commercial varieties, GRKML and GRKAL breeding lines, as well as in GR26 landrace.

It is conceivable that presence of alleles linked with agronomic traits as identified in other studies, does not necessarily imply the same for the materials examined in this study. Consequently, any association of these alleles to the expected phenotypes must be confirmed before the markers can be further used for breeding purposes. Thus, the alkaloid profile of the accessions was further examined.

### 2.4. Alkaloid Content Profile

**Figure 4:**
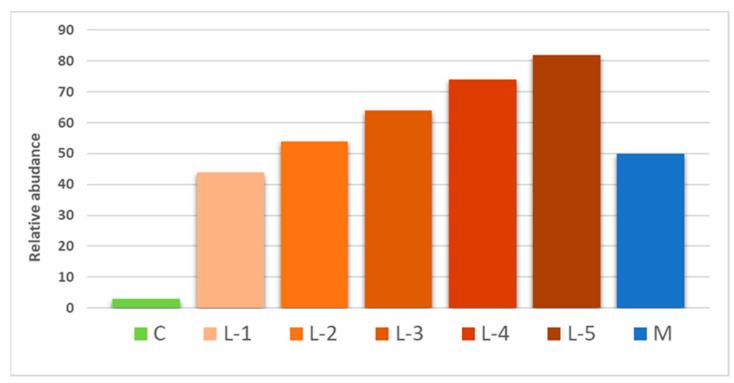
Relative alkaloid abundance in seed extracts of landraces grouped in five groups L-1 to L-5, according to the alkaloids abundance in comparison to cultivar Celina (C) and Multitalia (M).

The alkaloid profiling of accessions was investigated by Ultra-High Performance Liquid Chromatography coupled to Orbitrap High Resolution Mass Spectrometry Analysis (UHPLC-HRMS). Overall, the approach used allowed to tentatively annotate sixteen alkaloids, including angustifoline, lupinine, epilupinine, sparteine, etc. (online Supplementary Table S2) that are known to participate in the alkaloid profile of *Lupinus albus* L. seeds. A representative chromatogram of an accession of lupin seeds is presented in (online Supplementary Figure S1). Relative abundance of total alkaloids in landraces and two cultivars, namely Celina and Multitalia, is shown in Figure 4. Landraces are grouped in five groups (L-1, L-2, L-3, L-4 and L-5) according to their alkaloid abundance in comparison to the Celina cultivar that is a “sweet” variety exhibiting the lowest alkaloid content. It is noteworthy that Multitalia is a known bitter cultivar. Taken into account the alkaloid profile of the examined accessions we may conclude that the sole presence of the “sweet” *pauper* marker does not necessarily predict for a low alkaloid profile.

Interestingly the partitioning of the 16 alkaloid compounds assessed in each sample varied between accessions, indicating that each alkaloid biosynthesis product may accumulate in different amounts in different genotypes (online Supplementary Figure S2). Furthermore, landraces GR24, GR25, GR27, and GR28 with spotted seeds had no higher alkaloid content compared to those with white-yellowish seeds. Further studies implementing more specific markers of alkaloid biosynthesis pathways are required to complement systematically the seed alkaloid profile and the genetic alleles configuration of the genotype. Moreover, the partitioning of the 16 alkaloids in the metabolic profile of landraces is quite diverse indicating that a more thorough assessment of critical alleles that impart accumulation of alkaloids is necessary for an integrated assessment of valuable germplasm that could be underscored in the sole absence of the *pauper* allele.

## 3. Discussion

Genetic diversity of white lupin genetic resources in Greece was assessed using morphological and molecular markers. This is the first report for molecular characterization of local white lupin germplasm in a country that is considered as a center of genetic variation for this crop.

Seed morphological characteristics were selected because heritability of seed morphological traits and ease of access to seeds may provide a reasonable alternative to estimate the distribution of genetic variation [49]. Considerable variation was recorded for TSW, seed size, and coloration while less variation could be detected for circularity. Clustering of genotypes formed subclades consisted solely of landraces, although some landraces were partitioned with the cultivars. Other studies indicate high level of polymorphism for selected morphological traits in white lupin, while generally these characters are not very informative for landrace genetic diversity studies. Wide range of variation was recorded for proportion of pod walls and other pod components in 325 ecotypes originated from 17 countries of the Mediterranean region and north Africa. The variation was related with the country of origin of the accessions since Egyptian ecotypes had the lowest proportions of pod walls while the Greek and Italian ecotypes had the highest [50]. Analysis of Ethiopian landraces indicated the existence of high level of polymorphism for different agronomic traits and nutrient contents of grain [44]. In an extensive study of 35 Spanish white lupin accessions estimating variation for 50 quantitative and 51 qualitative characters, described in the literature as highly heritable, only a small number of three quantitative and one qualitative parameters were variable enough to provide good separation of the accessions [51].

It is conceivable that as valuable as they may be, agronomic traits and morphological characteristics as genetic markers are of limited resolution power for genetic diversity assessment, compared with molecular markers. In this study, molecular polymorphism was considerable. Relatively moderate to high PIC values, indicating a high level of genetic diversity in the germplasm assessed. The markers were suitable for discrimination of most landraces that clustered apart from the commercial cultivars indicating a unique diversity. Specifically, landraces were grouped into two clusters with those originated from Andros island forming a distinct group representing the wild *graecus* form of lupin, while all the others were grouped together regardless of geographic origin. Interestingly, all the commercial cultivars were grouped in one cluster.

Molecular markers have been proved valuable tools for germplasm diversity studies in many plant species. Yet, limited number of studies on white lupin molecular diversity are cited in the literature. To the best of our knowledge, genetic diversity using molecular markers has been estimated for local germplasm from Ethiopia [42,52] and Egypt [53]. Our results are consistent with the previous studies indicating high level of landrace accession polymorphism. Landraces from Ethiopia were highly polymorphic [43] and formed a distinct and separate grouping from Australian cultivars, breeding lines, and European cultivars, which cluster together, probably revealing their pedigree and breeding history [52]. Further development of molecular markers and application in genetic diversity studies could enhance our understanding of white lupin genetic resources and promote their use in modern breeding.

The Greek landscape is characterized by a mountainous terrain, with numerous peninsulas and islands, and is considered a hotspot of *L. albus* diversity. Such isolated regions, justify divergence of local landraces, explaining the finding that landraces from Andros and Lemnos islands and from Mani peninsula are clustered separately from the cultivars examined. Additionally, landraces from Andros island are distinctly diversified from the rest accessions, implying that Andros constitutes a genetic pool of unique genes, potentially useful for white lupin breeding.

Eventual exchange of landraces between Greek farmers and dispersal of their cultivation farther than their local point of origin may explain landrace sub-clusters on the dendrogram (Figure 2). It is also a common practice for smallholder farmers to use a portion of the seed yield for the next growing season, exclusively, or in mixtures with high yielding cultivars. Long periods of recycling the seed of a commercial variety, along with potential adaptation pressure and selection by the farmers, promote the generation of new variability, resulting in new landraces that are well adapted to the specific geoclimatic conditions and to their traditional management and uses [38,54,55]. Moreover, potential gene flow from neighboring white lupin cultivar crops could justify that landraces appear genetically in close proximity with commercial germplasm.

Quinolizidine alkaloids are predominantly found in high levels in natural populations and landraces, making lupin a repulsive choice for lambs and goats, that feed on pasture [56]. That justifies the presence of white lupin natural populations throughout uncultivated lands, that shelter underutilized genetic diversity. QA biosynthesis occurs in the vegetative upper part of the plant, and they are subsequently transported to the seeds. Modern breeding strategies focus on creating elite cultivars, with low alkaloid content (below 0.02% of the total dry weight) [15], as well as cultivars that retain QAs in vegetative tissues, thus producing hardy plants with “sweet” seeds [15]. Their biosynthesis is regulated by five different loci, with *pauper* being thoroughly investigated thus far, on account of its contribution to QA synthesis regulation [38,48,57]. Regarding the accessions under examination, the “sweet” *pauper* marker was detected in all cultivars, in breeding lines GRKML and GRKAL, and in GR26 landrace. Absence of the “sweet” *pauper* marker from the vast majority of the landraces is in accordance with their high QA content. However, presence of the marker in high QA genotypes, like the cultivar “Multitalia”, makes this marker a weak predictor of low QA content with broad applicability.

In Mediterranean farming systems, white lupin is considered a winter crop and sowing takes place in autumn, in order for the crop to take advantage of the late winter rainfalls. Thus, sufficient seed filling is succeeded before the early dry spells that occur in May, which have been more frequent and severe in Mediterranean ecosystems, due to the climate change [24]. Early flowering is considered to be an effective stress escape mechanism, in that it promotes fulfillment of the plants biological cycle, prior to terminal drought stress [46,58]. Additionally, genotypes non-responsive to vernalization, suggest crops suitable for spring sown cultivation, in northern regions, with long lasting winter. While white lupin germplasm native to the Balkan peninsula has been previously characterized as vernalization responsive [59,60], among the local landraces under investigation, GR3, GR7, GR12, GR16, GR19, GR21, and GR26 hold both *SEPALLATA 3* and *FLOWERING LOCUS T* early flowering alleles, implying a vernalization independence-promoting regional microclimate at the collection sites [31,60]. Moreover, the early flowering allele of *SEPALLATA 3* was detected in all commercial varieties, except for Ulysse. The inability of FTa1 primer set to hybridize in three landraces from Andros island and in cultivars Frieda and Celina, suggest contingent mutations located at the primer binding site, in the genomes of those accessions (Table 2). The *GIGANTEA* early flowering promoting allele was detected only in GR5, GR10, and Frieda cultivar. However, further experimentation on the aforementioned landraces is needed, in order to examine the predictive power of those molecular markers, and their potential implementation in white lupin breeding programs.

Alleles that confer resistance to anthracnose, were previously found only in Ethiopian landraces, which are distinctly related to European improved germplasm [42]. Resistance-conferring alleles, located in antr04_1/antr05_1 and antr04_2/antr05_2 loci, were also detected in the Greek landraces examined, with GR23 and GR25 from Andros, having both of them in their genome (Table 2). Summarizing these results, molecular markers linked to important agronomic traits have been identified in white lupin germplasm from the Greek rural areas. It will be significant to confirm the association of these markers to the relevant plant phenotype in further studies. This will be crucial for the utilization of the relative landraces when is necessary (e.g., stacking alleles present in one landrace), as sources of the responsible alleles in lupin breeding.

## 4. Materials and Methods

### 4.1. Plant Material

**Figure 5:**
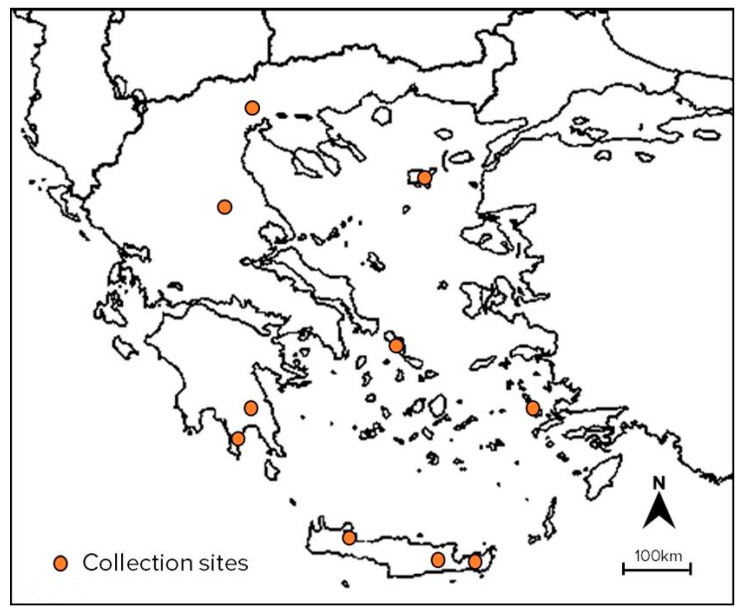
Map of collection areas of white lupin accessions used in the study (Google, n.d.).

Seeds of 28 landraces were obtained by local farmers and collection expeditions in rural areas throughout the Greek territory (Figure 5). Four experimental breeding lines were provided by the Institute of Plant Breeding and Genetic Resources of the Hellenic Agricultural Organization-DEMETER. Thirteen commercial varieties were provided by local representatives of the seed companies. Details on plant material are given in Table 3.

### 4.2. Estimation of Genetic Diversity

#### 4.2.1. Seed Morphological Diversity Analysis

The Thousand Seed Weight (TSW) was calculated, based on the weight of 100 seeds in three independent replicates. Seed morphological diversity was estimated, by subjecting 10 seeds of each sample to examination according to standard criteria (IBPGR 1981) using image analysis as previously described [61]. Calculation of the Euclidean distance, Principal Component Analysis (PCA), UPGMA dendrogram, Wilks’ Lambda test and Multivariate Analysis of Variance (MANOVA) between all 45 samples were performed, using the XLSTAT software (Data Analysis and Statistical Solution for Microsoft Excel, Addinsoft, Paris, France 2017).

#### 4.2.2. PCR and SSR-HRM Analysis

For every accession, the molecular genetic diversity was assessed on bulked samples of 5 individuals. Genomic DNA was extracted from seeds, applying the QIAGEN DNeasy Plant Pro Kit (Qiagen, Hilden, Germany). The yield of the extracted DNA was estimated with Qbit 4 Fluorometer (Thermo Fisher Scientific, Waltham, MA, USA) and normalized to 5 ng/μL for downstream applications. Genotypic analysis was performed using 6 polymorphic microsatellite markers (online Supplementary Table S1), based on reports by Nelson et al. [62]. PCR amplification reactions and High-Resolution Melting Analysis were carried out on LightCycler^®^ 96 (Roche Diagnostics Gmbh, Mannheim, Germany), using KAPA HRM FAST PCR Kit (KAPA Biosystems, Wilmington, MA, USA), in a total reaction volume of 12 μL, containing 5 ng genomic DNA template, 1X KAPA HRM FAST Master Mix, 0.2 μM of each primer, 2.5 mM MgCl_2_, and PCR-grade H_2_O. The PCR amplification program constitutes an initial denaturation step at 94 °C/4 min, followed by 35 cycles of denaturation at 94 °C/30 s, primer annealing at 58 °C, and extension at 72 °C/40 s. The HRM step includes denaturation at 95 °C/60 s, annealing at 40 °C/60 s, gradual denaturation from 65 °C to 97 °C by 0.05 °C/s, and fluorescence detection 20 times per °C.

### 4.3. Analysis of Molecular Genetic Relationships

The samples were assigned to groups, according to the amplicons melting temperature (Tm), the shape of the normalized melting curves, and the difference plots, generated by the HRM analysis, with different groups denoting dissimilar genetic profiles, and a binary matrix was formed, for further analyses. Genetic distances between all 45 accessions were calculated based on Nei’s genetic distance [63], and a dissimilarity matrix was generated using GenAlEx 6.5, which was subjected to tree construction, under the Unweighted Pair Group Method Analysis (UPGMA), using MEGA X [64]. Additionally, Principal Coordinates Analysis (PCoA) was carried out on GenAlEx 6.5. The Polymorphic Information Content of the SSR markers was calculated according to Smith et al. (1997). Potential correlation of the genetic distances calculated by morphological and molecular diversity, was investigated through Mantel’s test. The association of the molecular markers to the seed morphological traits was estimated through multiple linear regression analysis, using IBM SPSS Statistics for Windows Version 24.0 (IBM Corp., Armonk, NY, USA).

### 4.4. Germplasm Molecular Characterization on Anthracnose Resistance, Vernalization Responsiveness amd Alkaloid Biosynthesis

Germplasm evaluation on agronomically important traits, was addressed by investigating for the presence of alleles, that control low alkaloid content, resistance to anthracnose and vernalization requirement, applying a set of recently developed, specifically designed dCAPS molecular markers (Table 4) [18,19,48]. PCR amplification reactions were carried out on Veriti Dx Thermal Cycler (Applied Biosystems^®^, Waltham, MA, USA), using KAPA Taq ReadyMix PCR Kit (Kapa Biosystems, Wilmington, MA, USA) in a total reaction volume of 15 μL, containing 20 ng genomic DNA, 1X KAPA Taq ReadyMix Mix, 0.2 μM of each primer, 1.5 mM MgCl_2_, and PCR-grade H_2_O. PCR amplification was performed under the following conditions: initial denaturation at 94 °C for 4 min, followed by 35 cycles of denaturation at 94 °C for 30 s, annealing for 30 s, extension at 72 °C for 30 s, and final extension at 72 °C for 4 min. In order to detect the presence of the target allele of each marker, all samples were subjected to post-PCR restriction enzyme digestion, following the appropriate protocol provided by New England BioLabs (Ipswich, MA, USA). The presence or absence of the target alleles was then verified by 3% agarose gel electrophoresis (1× TAE) on 100 volts for 40 min.

### 4.5. Extraction of Alkaloids and Profiling by HPLC-HRMS-Mass Spectrometry

Sample preparation was adopted from a recently published study [65], incorporating minor modifications. Briefly, a mixture of 0.5 g of pulverized seeds with a MeOH:H_2_O, 4:1 (*v*/*v*) solution (10 mL) containing 0.1% formic acid was vortex mixed (1 min), and then extracted with an ULTRA-TURRAX homogenizer (Ika T25, Staufen, Germany) for 4 min in total (4 rounds of 1 min, applying a 15 s break between each round) at 4 °C. Consequently, after centrifugation the supernatant was filtered with Nylon filters (0.22 μm), collected, and a 1:100 dilution was applied to furnish the final extract working solution. The latter was injected to the UHPLC-HRMS system (Ultra-High Performance Liquid Chromatography)—coupled to Q-Orbitrap High Resolution Mass Spectrometry.

Samples were analyzed with a Dionex Ultimate 3000 UHPLC system (Thermo Fisher Scientific, San Jose, CA, USA) linked to Q-Exactive Orbitrap HRMS (Thermo Fisher Scientific, San Jose, CA, USA). A Hypersil Gold UPLC C18 (2.1 × 100 mm, 1.9 μm) reversed phased column (Thermo Fisher Scientific, San Jose, CA, USA) was used for the separation that was maintained at 40 °C. Gradient elution of analytes was carried out with aqueous 0.1% (*v*/*v*) formic acid (A) and 0.1% (*v*/*v*) formic acid in acetonitrile (B). The gradient elution was: T = 0 min, 5% B; T = 3 min, 5% B, T = 21 min, 95% B, T = 23 min, 95% B, T = 24 min, 5% B; T = 30 min, 5% B. The flow rate was 0.22 mL/min and the injection volume 3 μL. The ionization was performed using heated electrospray ionization (HESI), in the positive ion mode. The conditions for the HRMS were set as follows: capillary temperature, 350 °C; spray voltage, 2.7 kV; S-lense Rf level, 50 V; sheath gas flow, 40 arb. units; aux gas flow, 5 arb. Units; aux. gas heater Temperature, 50 °C. The analysis was performed in the full scan ion mode, applying a resolution of 70,000, with a mass range of 100–1200 *m*/*z* while acquisition of the mass spectra was performed in the centroid mode.

All reagents and chemicals used were of analytical grade. Acetonitrile (I), methanol (MeOH), and formic acid of LC-MS grade were obtained from Merck (Darmstadt, Germany). Ultra-pure water was produced from SG Milipore apparatus. Nylon filters (0.22 μm) were obtained from Macherey-Nagel (Dueren, Germany). 

Post-acquisition data analyses were performed using Compound Discoverer 2.1 software (Thermo Fisher Scientific, San Jose, CA, USA). The software was used to manipulate data and apply procedures of chromatographic processing region selection, application of baseline correction, peak detection, deconvolution, peak alignment, deisotoping and gap filling. For the putative annotation of the compounds a custom library was prepared based on the expected compounds of the genus of Lupinus using as a source the Dictionary of Natural Products applying a tolerance of 5 *m*/*z*.

## 5. Conclusions

Legumes are pivotal for the sustainability of farming and food systems, by promoting soil fertility and environmental protection, in addition to food security. White lupin represents an emerging crop, providing both agricultural and nutritional benefits, yet in demand of extensive breeding. This study highlighted the presence of highly variable Mediterranean landraces, concealing potentially valuable genetic loci for adaptation in the local conditions, important for lupin breeding. The landraces evaluated are considered bitter-seeded, as they possess high-alkaloid content and probably the relative allele(s) in their genome. Therefore, they require debittering processing prior to consumption. Further breeding, addressing the reduction of high-alkaloid seed content, could yield lupin seeds ready-to-use in food industry and in forage as well. Moreover, 8 landraces were identified harboring the *SEPALLATA 3* vernalization unresponsiveness-allele, as well as 2 landraces possessing both anthracnose-resistance alleles. These landraces could provide a significant genetic resource, to be harnessed in white lupin breeding programs. Expansion of white lupin cultivation to various hardiness zones, would promote legume reintroduction to the European farming systems, endorsing agriculture and environment sustainability in support of green farming.

## Figures and Tables

**Table 1:** Summary statistics of the seed morphological characters.

Variable	Observations	Minimum	Maximum	Mean	Std. Deviation
TSW (g)	45	70.8	968.2	357.2	116.7
Area (mm^2^)	45	628.0	2317.2	963.2	260.5
Perimeter (mm)	45	304.5	578.8	374.1	42.2
Circularity ^†^	45	0.076	0.090	0.085	0.004
Height (mm)	45	66.7	105.9	81.1	9.4
Width (mm)	45	82.3	140.5	102.8	12.2
Gray Value ^†^ (Min.)	45	0.000	102.	46.1	27.5
Gray Value ^†^ (Max.)	45	128.0	255.	205.6	34.8
Gray Value ^†^ (Mean)	45	70.4	166.6	136.1	28.2
Gray Value ^†^ (Median)	45	65.0	169.0	137.0	28.7
Integrated Density ^†^	45	576,855.0	23,398,105.0	6,649,080.0	6,775,359.0

^†^ Circularity is morphometric and gray value and integrated density are densitometric parameters that have no units.

**Table 2:** Allele scoring on anthracnose resistance and early flowering. Symbol ”+” indicates the presence and “−“ indicates the absence of the marker alleles, that confer anthracnose resistance and early flowering, “*” indicates inconclusive allele detection.

Accession	Anthracnose Resistance		Early Flowering		
	TP222136	TP338761	GI-F1	SEP3-F1	FTa1-F1
GR1	−	+	−	−	+
GR2	−	+	−	−	+
GR3	−	−	−	+	+
GR4	+	−	−	−	+
GR5	−	−	+	−	+
GR6	−	−	−	−	+
GR7	+	−	−	+	+
GR8	+	−	−	−	+
GR9	+	−	−	−	+
GR10	+	−	+	−	+
GR11	+	−	−	−	+
GR12	−	−	−	+	+
GR13	+	−	−	−	+
GR14	+	−	−	−	+
GR15	+	−	−	−	+
GR16	+	−	−	+	+
GR17	+	−	−	−	+
GR18	+	−	−	−	+
GR19	−	−	−	+	+
GR20	−	−	−	*	+
GR21	+	−	−	+	+
GR22	+	−	−	−	+
GR23	+	+	−	−	+
GR24	+	*	−	*	*
GR25	+	+	−	−	*
GR26	+	−	−	+	+
GR27	+	−	−	−	+
GR28	−	*	−	−	*
GR29	−	−	−	+	+
GRKML	+	−	−	−	+
GRKAL	+	−	−	−	+
GRKCA	+	−	−	−	+
Energy	+	−	−	+	+
Magnus	−	−	−	+	+
Orus	−	−	−	+	+
FAS	−	−	−	+	+
Estoril	−	−	−	+	+
Ulysse	−	−	−	−	+
Sulimo	−	+	−	+	+
Figaro	−	−	−	+	+
Multitalia	−	−	−	+	+
Tennis	−	−	−	+	+
Amiga	−	−	−	+	+
Frieda	+	−	+	+	*
Celina	+	−	−	+	*

**Table 3:** Details on genotype, collection sites and origin, sample source, and sample type of the plant material.

Accession	Collection Site/Origin	Genotype	Sample Type	Sample Site
GR1	Crete	Landrace	Cultivated area	Cultivated
GR2	Crete	Landrace	Cultivated area	Cultivated
GR3	Crete	Landrace	Farm storage	Cultivated
GR4	Crete	Landrace	Cultivated area	Cultivated
GR5	Leros	Landrace	Farm storage	Cultivated
GR6	Leros	Landrace	“	“
GR7	Lemnos	Landrace	“	“
GR8	Lemnos	Landrace	“	“
GR9	Lemnos	Landrace	“	“
GR10	Lemnos	Landrace	“	“
GR11	Lemnos	Landrace	“	“
GR12	Lemnos	Landrace	“	“
GR13	Macedonia	Landrace	“	“
GR14	Macedonia	Landrace	“	“
GR15	Mani	Landrace	“	“
GR16	Mani	Landrace	“	“
GR17	Mani	Landrace	“	“
GR18	Mani	Landrace	“	“
GR19	Crete	Landrace	“	“
GR20	Crete	Landrace	“	“
GR21	Lakonia	Landrace	“	“
GR22	Lakonia	Landrace	“	“
GR23	Andros	Landrace	Uncultivated disturbed area	Wild
GR24	Andros	Landrace	Uncultivated and undisturbed area	Wild
GR25	Andros	Landrace	“	“
GR26	Andros	Landrace	Uncultivated and disturbed area	Wild
GR27	Andros	Landrace	“	“
GR28	Andros	Landrace	“	“
GR29	Thessaly	Breeding Line	Agricultural institute	Breeder’s line
GRKML	Thessaly	Breeding Line	“	“
GRKAL	Thessaly	Breeding Line	“	“
GRKCA	Thessaly	Breeding Line	“	“
Energy	France	Cultivar *	Company	Cultivated
Magnus	France	Cultivar	“	“
Orus	France	Cultivar	“	“
FAS	EU	Cultivar	“	“
Estoril	Portugal	Cultivar	“	“
Ulysse	France	Cultivar	“	“
Sulimo	France	Cultivar	“	“
Figaro	France	Cultivar	“	“
Multitalia	Italy	Cultivar	“	“
Tennis	Italy	Cultivar	“	“
Amiga	France/Czech Republic	Cultivar	“	“
Frieda	Germany	Cultivar	“	“
Celina	Germany	Cultivar	“	“

* According to the European Plant Variety Database (PVD, 2021).

**Table 4:** Molecular markers used for germplasm characterization on agronomic traits.

Trait	Molecular Marker	Validated Enzyme	Phenotype: Allele (bp)
QA content	LAGI01_35805_F1_R1	BclI	low QA content: 197
			high QA content: 108, 89
Anthracnose resistance	TP222136	CviKI-1	Anthr. susceptible: 168, 27, 15
			Anthr. resistant: 183.27
	TP338761	SchI	Anthr. susceptible: 83, 28
			Anthr. resistant: 64, 47
Vernalization requirement	GI-F1	AciI	vernalization unresponsive: 127, 30
			vernalization responsive: 157
	FTa1-F1	-	vernalization unresponsive: 2036
			vernalization responsive: 1353
	SEP3-F1	TaqI	vernalization unresponsive: 122, 23
			vernalization responsive: 145

## Data Availability

The data that support the findings of this study are available from the corresponding author upon reasonable request.

## Supplementary Materials

The following are available online at https://www.mdpi.com/article/10.3390/plants10112403/s1, Table S1: Primers of molecular markers used for estimating the genetic diversity, Table S2: Sixteen alkaloids were tentatively annotated in all the white lupin seeds extracts after the UHPLC–HRMS (Orbitrap) analysis, using a custom library based on the genus Lupinus and applying tolerance of 5 *m*/*z*, Figure S1: Representative chromatogram of a white lupin seed extract sample after the UHPLC–HRMS (Orbitrap) analysis, in the positive ion mode, Figure S2: Alkaloids chemical profile of selected seed extracts, Figure S3: Representative seeds of the GR24 landrace, Figure S4: Representative seeds of the GR25 landrace, Figure S5: Representative seeds of the GR27 landrace, Figure S6: Representative seeds of the GR28 landrace.
