# Supplementary material for: Mediterranean White Lupin Landraces as a Valuable Genetic Reserve for Breeding
Source: Plants (Basel). 2021 Nov 7;10(11):2403. doi: 10.3390/plants10112403 (PMC8619254; doi:10.3390/plants10112403)
Supplement: Supplementary file 1 [file plants-10-02403-s001.zip › Figure S1.pdf]

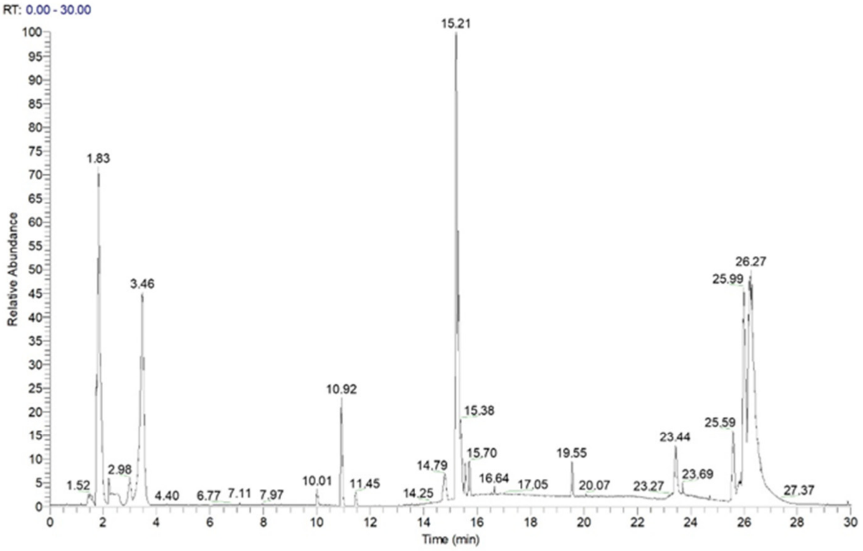

Figure S1. Representative chromatogram of a white lupin seed extract sample after the UHPLC–HRMS (Orbitrap) analysis, in the positive ion mode
