# Supplementary material for: Mediterranean White Lupin Landraces as a Valuable Genetic Reserve for Breeding
Source: Plants (Basel). 2021 Nov 7;10(11):2403. doi: 10.3390/plants10112403 (PMC8619254; doi:10.3390/plants10112403)
Supplement: Supplementary file 1 [file plants-10-02403-s001.zip › Table S1.pdf]

Table S1. Primers of molecular markers used for estimating the genetic diversity.

Marker	Forward Primer (5′-3′)	Reverse Primer (5′-3′)	Tm	PIC value
LSSR06a	GTTGTTTTGGGACAACACCC	AAAACCCGAACCTGTGTAGC	54ºC	0,8
LSSR07	GGATCAGGTGCTTTCAGTCTTG	AACCTCATCGAGTGTGAGACTGTAC		0,7
LSSR10	CGTCATCCATCAATGTTTGG	TTAAGGAAACACTGGCCCAT		0,2
LSSR11	TCCCTTGCTCTTTTCCTCAC	CCGTTTAGCACATTGGCAC		0,6
LSSR14	GGTGACCCTCACCAGAACAT	GGTCCTTTGATGATGGTGCT		0,5
LSSR41	TCAAGGGTGCTCAGTATGGG	TCATCCTTTCCCTCCAAAGA		0,8
LAGI01-35805 _F1_R1	TGGCATCACTGAAAATTGAGATGA	CTTTGAGAGGGCTTGTTTGAG	64ºC	0,4
TP222136	CTTCACCCAGTCTCTATCTGCAC	AATGAGCATGCTTAATCTTGTTGCA	63ºC	0,5
TP338761	TCCTTGAGAGAATCCAAGCTGC	CTACAATGCACACGAGATTGCC	60ºC	0,3
GI-F1	TCTAGAGCAGGTAATCCAGCAAC	TTGCTAATGGTGCTGGTGTTATATTAAA	56ºC	0,1
FTa1-F1	AAGGGTCTTGGATTTTCATAACTTACA	AGCAACTACTGGGCCTAGT	62ºC	0,2
SEP3-F1	TATTTAAGGATGAAGCACCA	TTTTTATCATTTCATTTTCTTCG	56ºC	0,5

* PIC value: Polymorphic Information Content
