# Supplementary material for: Mediterranean White Lupin Landraces as a Valuable Genetic Reserve for Breeding
Source: Plants (Basel). 2021 Nov 7;10(11):2403. doi: 10.3390/plants10112403 (PMC8619254; doi:10.3390/plants10112403)
Supplement: Supplementary file 1 [file plants-10-02403-s001.zip › Table S2.pdf]

Table S2. Sixteen alkaloids were tentatively annotated in all the white lupin seeds extracts after the UHPLC–HRMS (Orbitrap) analysis, using a custom library based on the genus Lupinus and applying tolerance of 5 *m/z.*

**Alkaloids and derivatives Putative annotation**	**Molecular Formula**	**Monoisotopic Mass (Da) Experimental**	**tR* (min)**	**Mass Difference (ppm)**
(+)-Epilupinine N-Oxide	$C_{10}H_{19}NO_2$	185.1416	1.82	3.08
(-)-N-methyl-Angustifoline	$C_{15}H_{24}N_2O$	248.1887	1.86	1.55
Lupinine	$C_{10}H_{19}NO$	169.1467	1.75	3.45
Oxymatrine	$C_{15}H_{24}N_2O_2$	264.1836	2.85	1.4
Albine	$C_{14}H_{20}N_2O$	232.1576	4.26	2.52
4ξ-O-Angeloyl-4-Hydroxylupanine	$C_{20}H_{30}N_2O_3$	346.2255	9.4	1.17
(+)- Epilupinine	$C_{10}H_{19}NO$	169.1467	2.22	3.45
(+)-Angustifoline	$C_{14}H_{22}N_2O$	234.1732	6.64	2.29
(R)-Ammodendrine	$C_{12}H_{20}N_2O$	208.1576	7.36	2.81
Sparteine	$C_{15}H_{26}N_2$	234.2096	3.87	2.35
Anagyrine	$C_{15}H_{20}N_2O$	244.1576	1.76	2.4
13α-Tigloyloxy Multiflorine	$C_{20}H_{28}N_2O_3$	344.21	9.50	1.61
7-Hydroxysparteine; 6-Epimer	$C_{15}H_{26}N_2O$	250.2043	1.76	1.34
O-2-Methylbutanoyl 13-Hydroxylupanine	$C_{20}H_{32}N_2O_3$	348.2412	9.71	1.31
Benzoyl 13-Hydroxylupanine	$C_{22}H_{28}N_2O_3$	368.2099	10.06	1.24
Alkaloid LV-2	$C_{15}H_{24}N_2O_2$	264.1837	1.92	1.78
