# Supplementary figures and images for: Mediterranean White Lupin Landraces as a Valuable Genetic Reserve for Breeding
Source: Plants (Basel). 2021 Nov 7;10(11):2403. doi: 10.3390/plants10112403 (PMC8619254; doi:10.3390/plants10112403)

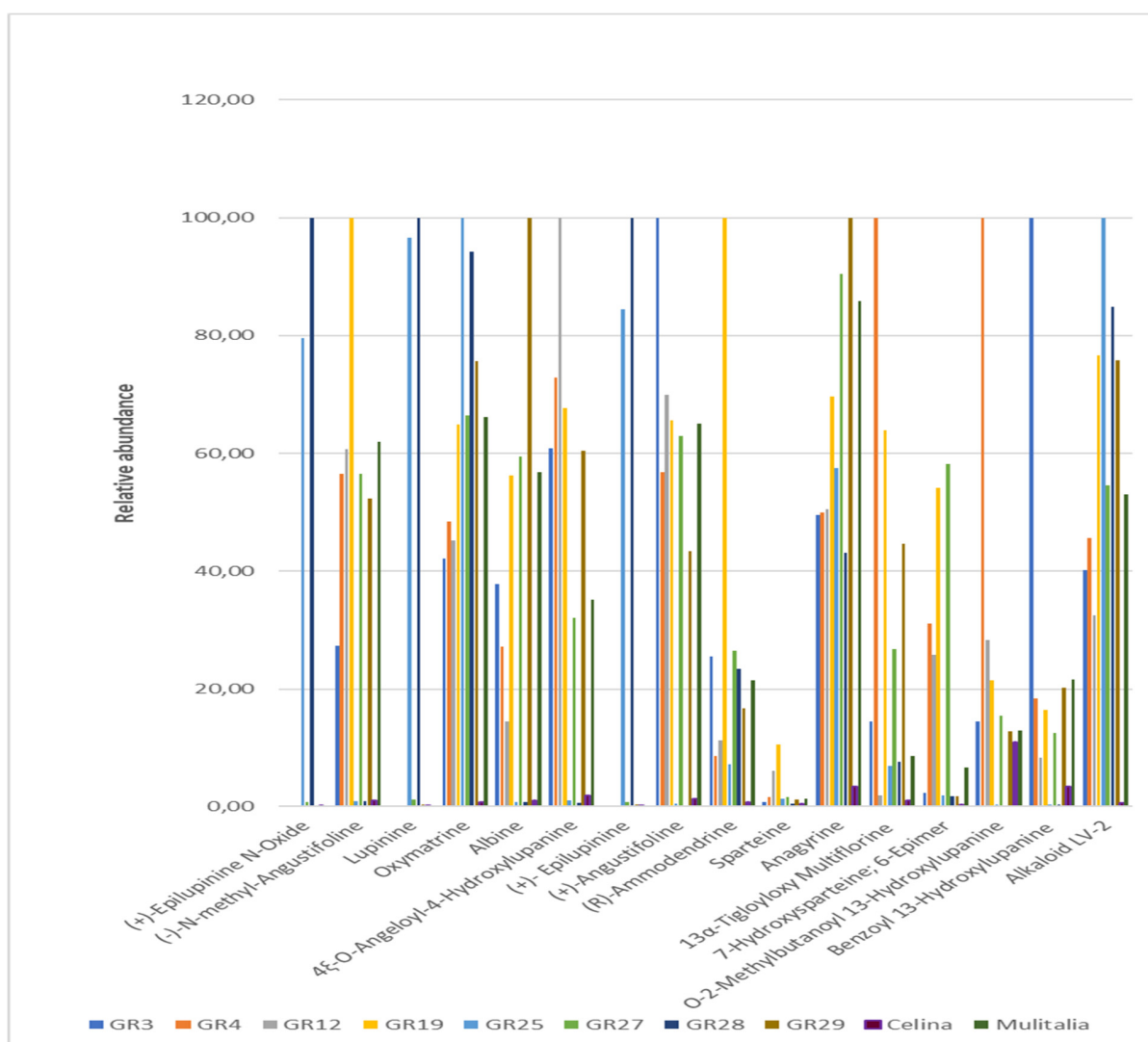

**Figure S2.** Alkaloids chemical profile of selected seed extracts.

Supplement: Supplementary file 1 [file plants-10-02403-s001.zip › Figure S2.pdf]

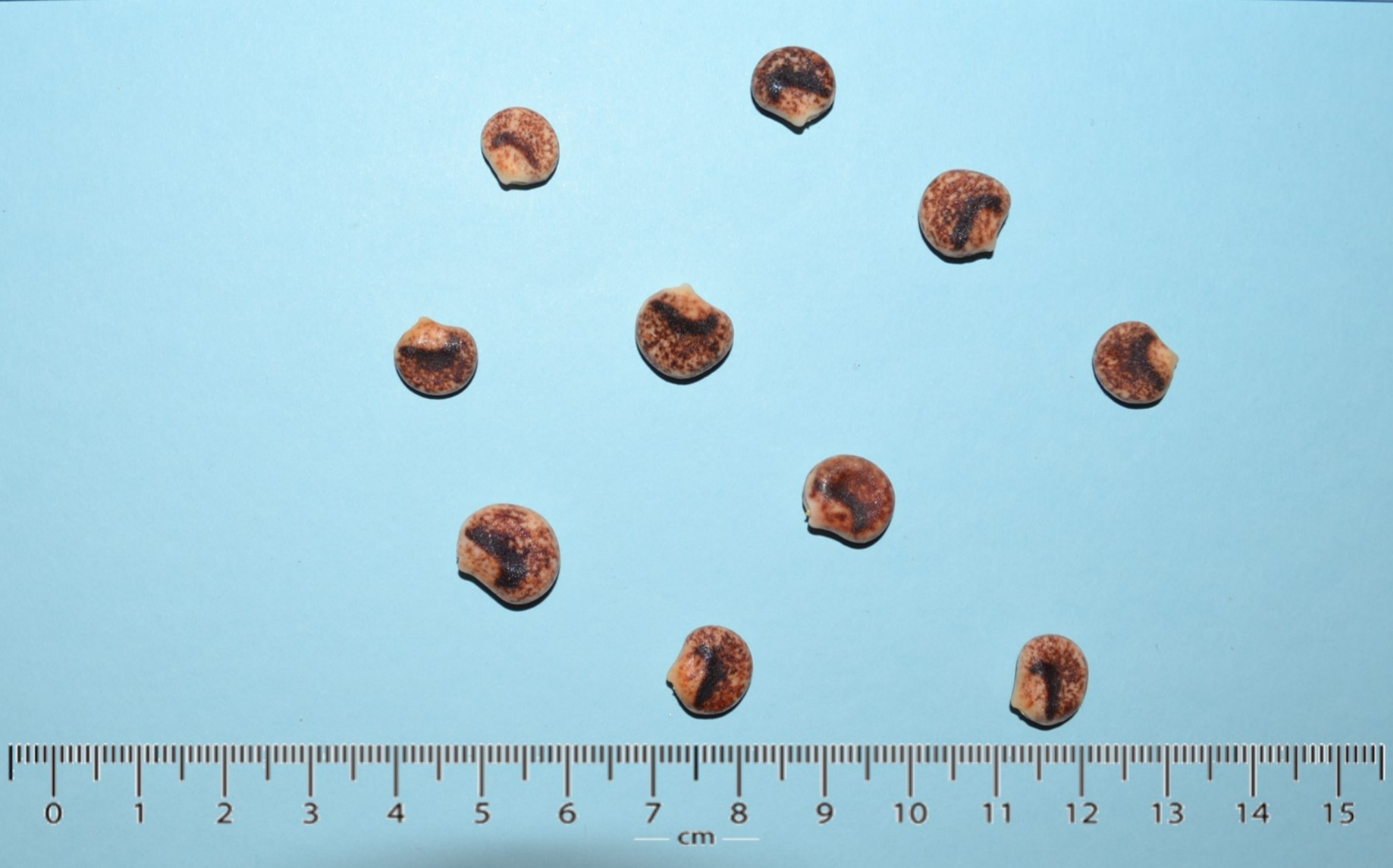

Supplement: Supplementary file 1 [file plants-10-02403-s001.zip › Figure S3 GR24 seeds.pdf]

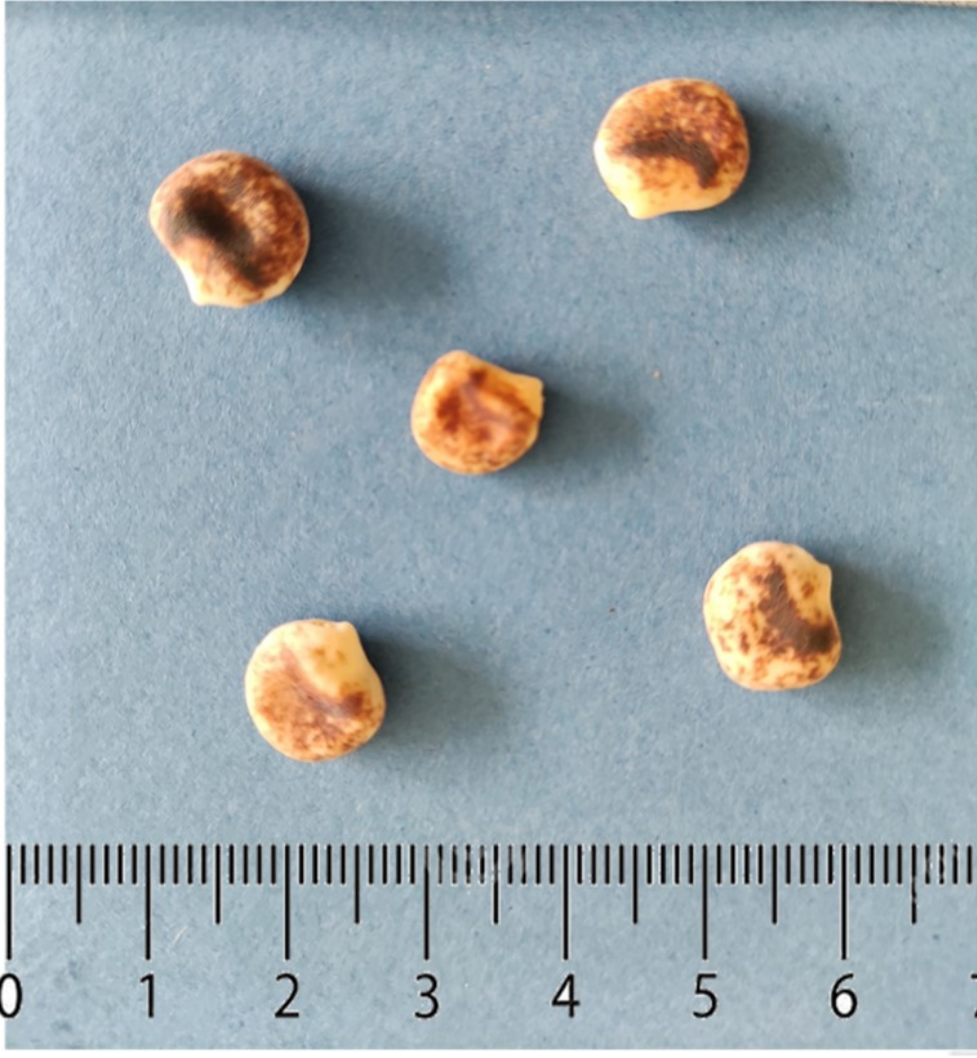

Supplement: Supplementary file 1 [file plants-10-02403-s001.zip › Figure S4 GR25 seeds.pdf]

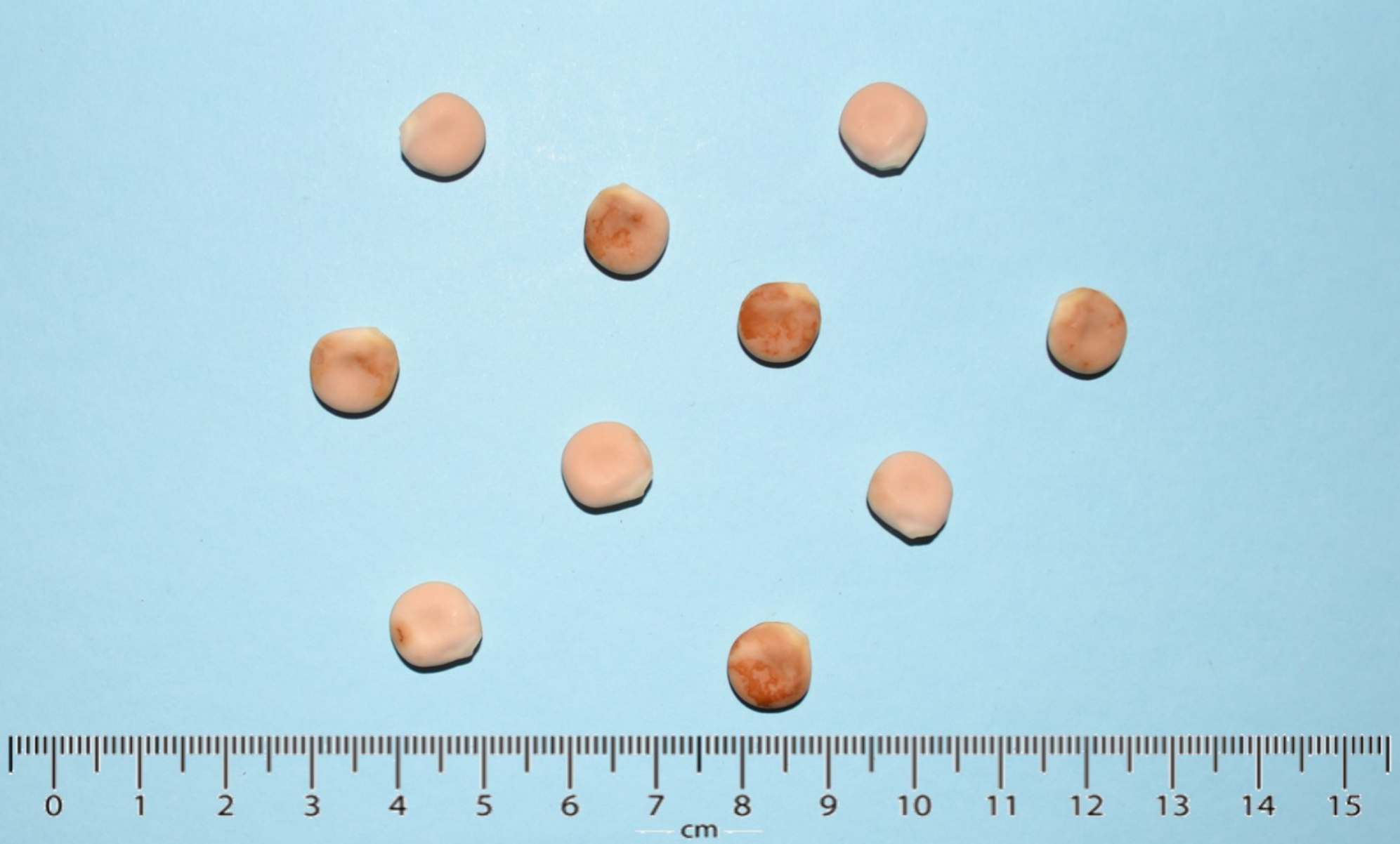

Supplement: Supplementary file 1 [file plants-10-02403-s001.zip › Figure S5. GR27 seeds.pdf]

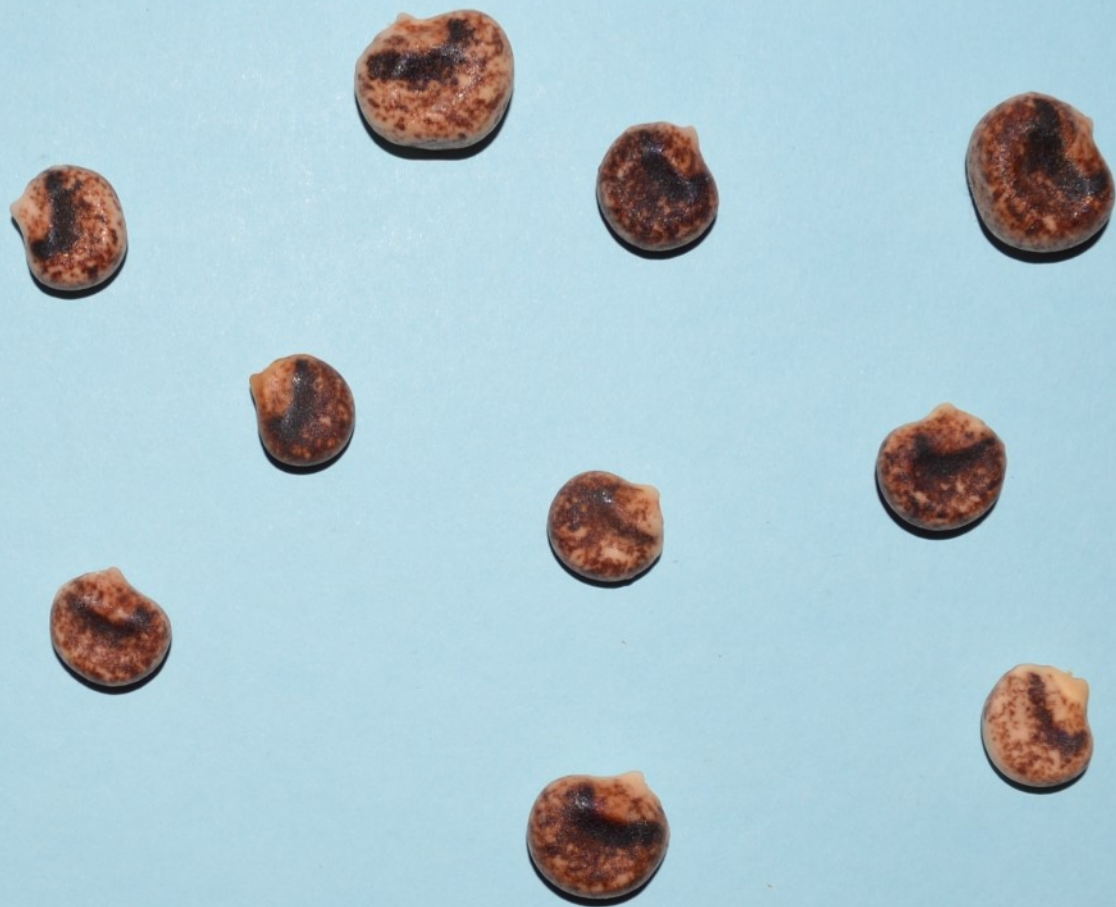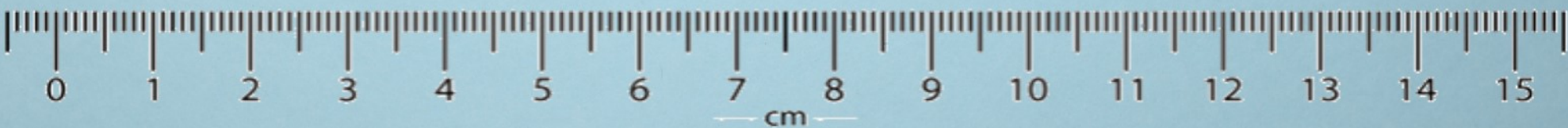

Supplement: Supplementary file 1 [file plants-10-02403-s001.zip › Figure S6. GR28 seeds.pdf]
